# Evaluation of a Bayesian inference network for ligand-based virtual screening

**DOI:** 10.1186/1758-2946-1-5

**Published:** 2009-04-29

**Authors:** Beining Chen, Christoph Mueller, Peter Willett

**Affiliations:** 1 Krebs Institute for Biomolecular Research, Departments of Chemistry and of Information Studies, University of Sheffield, Sheffield, S10 2TN, UK

## Abstract

**Background:**

Bayesian inference networks enable the computation of the probability that an event will occur. They have been used previously to rank textual documents in order of decreasing relevance to a user-defined query. Here, we modify the approach to enable a Bayesian inference network to be used for chemical similarity searching, where a database is ranked in order of decreasing probability of bioactivity.

**Results:**

Bayesian inference networks were implemented using two different types of network and four different types of belief function. Experiments with the MDDR and WOMBAT databases show that a Bayesian inference network can be used to provide effective ligand-based screening, especially when the active molecules being sought have a high degree of structural homogeneity; in such cases, the network substantially out-performs a conventional, Tanimoto-based similarity searching system. However, the effectiveness of the network is much less when structurally heterogeneous sets of actives are being sought.

**Conclusion:**

A Bayesian inference network provides an interesting alternative to existing tools for ligand-based virtual screening.

## Background

Virtual screening is the name given to a range of computational techniques for searching a chemical database to assess the probability that each molecule will exhibit activity against a specified biological target [1]. These techniques can be used to enhance the effectiveness of lead-discovery programmes since they ensure that only those molecules with reasonable *a priori *probabilities of activity are considered for conventional biological screening.

The virtual screening approaches that can be used in any particular circumstances depend principally upon the amounts and types of data that are available [2-7]; here we focus on ligand-based approaches, of which there are three main classes. If just a single active molecule is available, such as a competitor's compound or a natural product, then similarity searching can be used, in which a database is ranked in decreasing order of similarity to the known active structure. If several structurally related actives have been identified then pharmacophore mapping can be carried out to ascertain common patterns of features; these patterns are then searched using a 2D or 3D substructure search procedure. If it is not possible to identify a common pharmacophore, as often occurs with heterogeneous sets of actives, and if significant numbers of both active and inactive molecules are available, then these can be used as training data for a machine learning system.

The simplest, and probably the most widely used, technique for virtual screening is similarity searching. Here, the database structures are ranked in decreasing order of similarity with the active, user-defined reference structure, with the expectation that the nearest neighbours will exhibit the same activity as the reference structure. There is a huge literature associated with the measurement of molecular similarity [8-16]. The most common approach, which we study in this paper, uses molecules characterised by 2D fingerprints, with the similarity between a reference structure and a database structure calculated using an association coefficient such as the Tanimoto coefficient [1,8]. There are, however, other ways in which the structural information encoded in a fingerprint can be used, and in this paper we report a detailed analysis of one way in which this can be done. Specifically, we report the use of a Bayesian inference network for ligand-based virtual screening and compare its screening performance with a conventional, Tanimoto-based searching system.

## Results

### The algorithm

A Bayesian inference network (hereafter BIN) is a tool that permits the computation of the probability that an event will occur, allowing for the fact that this chosen event can be dependent on other events occurring. Our interest has been spurred by work in information retrieval, where BINs have been used to rank textual documents in decreasing probability of relevance to a user-defined query statement. In particular, Croft and his collaborators have used a BIN as the basis for the InQuery retrieval system [17-20] and for subsequent work on the use of language models in information retrieval [21,22]. To provide the necessary background, we first describe the operation of a BIN when it is used for textual information retrieval, and then show, in the next section, how simple modifications enable it to be used for similarity-based virtual screening.

**Figure 1:**
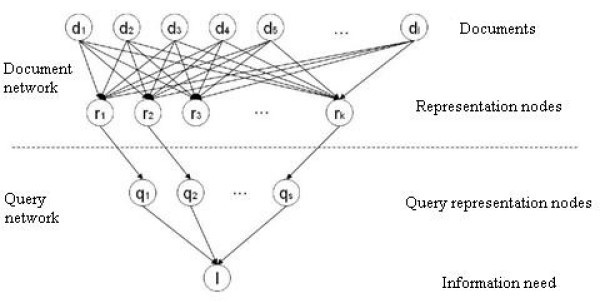
**Use of a Bayesian inference network for information retrieval**. In the chemoinformatics context, the Document network describes the structures of the database molecules, and the Query network describes the reference structure that is to be searched against this database.

The BIN in InQuery is a directed-acyclic dependency graph (DAG) in which the nodes represent propositional variables or events, which can be true or false, and in which the edges represent relationships between the propositions, i.e., an edge is drawn between two nodes if there is a conditional relationship between them. For example if the node *p *causes *q*, a conditional dependence between them exists, denoted by P(*q*|*p*). Associated with each relationship in the DAG is the degree of belief, which measures the magnitude of the influence of a parent node on a child node. The degrees of belief are stored in a storage-efficient manner in a data structure called a canonical link matrix [17]. An example of a simple BIN is shown in Figure 1, which consists of two parts. The document network represents the database that is to be searched, and hence needs to be generated just once when the database is created. The query network represents the query that is to be searched against the database, and can be regarded as an inverted DAG connected to the document network. Based on the connectivity and the interactions between the nodes, the network can then be evaluated by calculating the probabilities throughout the network starting with the root nodes.

The root nodes in Figure 1, denoted by *d*_*l*_, represent the event that a document is observed. Associated with each such event is a probability, or belief. The representation nodes (*r*_*k*_) represent the event that a particular indexing term (e.g., a keyword, a phrase or a thesaural term) is observed. The associated probability is called a belief function, *bel*(*r*_*k*_), and much of the BIN research in information retrieval has focused on belief functions that take account of the weighting of index terms. The weights that have been developed are based principally on two ideas: term weighting, where the importance of a term is proportional to the frequency with which a term occurs in an individual document or query; and inverse document frequency weighting, where the importance of a term is inversely proportional to its frequency of occurrence within the database as a whole [23-25].

The first layer in the query network contains the query nodes, each of which describes an operator that expresses constraints between the words in the retrieved documents. Examples of such operators used in the InQuery system of Croft *et al*. [17-19] include: Boolean AND, OR and NOT; weighted AND; maximum, sum and weighted sum of the beliefs. The root of the query network, *I*, represents the information need: this node combines all the information from its parent nodes into a single value. To evaluate the Bayesian network, the state of each document in turn is set to true (and the state of all the other document nodes set to false) and the belief then propagated through the network by calculating the posterior probabilities for each node. The posterior probability of the information need node then represents the conditional probability of that document being relevant to the given query. The procedure is repeated for each of the documents and the database then ranked in order of decreasing probability of relevance to the query.

### The implementation

We have noted previously the close relationship that exists between many of the methods that are used for textual information retrieval and for the processing of chemoinformatics databases [16,26], and it is this relationship that occasioned our initial interest in the application of BINs to virtual screening. Specifically, we suggest here that the BIN model outlined above can be applied to similarity-based virtual screening by replacing the index terms, documents, query and conditional probability of relevance in Figure 1 by substructural fragments, database structures, reference structure and conditional probability of activity, respectively. In this way, we can compute the conditional probability of activity for each database structure, and hence rank the database in decreasing order of these probabilities. While there are close analogies between the two application domains, it is worth noting at this point one considerable difference. In information retrieval, the documents in a large text database can contain many hundreds of thousands, or millions, of distinct terms (even if attention is restricted to individual words) whereas the query statement will contain only a very small number of these (often just two or three words in Web searches); in similarity searching, both the reference structure and the database structures are represented in the same way by a fingerprint containing a few hundred or a few thousand elements (1024 in the fingerprints studied here) and there is thus a greater degree of overlap in the database and query representations.

As noted in the previous section, the belief function, *bel*(*r*_*k*_), plays an important part in any BIN, and we have used four different belief functions here. These have all been used previously in information retrieval to model information about the occurrences of textual keywords, but have been modified here to model information about the occurrences of substructural fragments. The belief function that was originally used in InQuery is:

Here, *db *is the default belief, *tf*_*rk*, *dj *_is the frequency of occurrence of the fragment *r*_*k *_in molecule *d*_*j*_, max *tf*_*dj *_is the maximum frequency of occurrence in molecule *d*_*j*_, *df*_*rk *_is the number of molecules containing *r*_*k *_and *N *is the total number of molecules. An alternative, but closely related, belief function that has been extensively used in the TREC series of text retrieval experiments [27] is one developed on the OKAPI project:

Here, |*d*_*j*_| is the size (in terms of number of fragments) of the molecule *d*_*j *_and |*D*_*avg*_| is the average size of all the molecules in the database. Finally, Metzler and Croft have used belief functions, called smoothing functions, from studies of language modeling, which is a formal probabilistic framework for studies in speech recognition and statistical machine translation [21]. The Jelinek-Mercer smoothing function was found to be the most effective for information retrieval and is:

Here λ is a constant and *cf*_*rk *_is the sum of the frequencies of occurrence for the fragment *r*_*k *_in the database:

Details of these belief functions are provided in the cited literature, and we have used all three of them in our experiments: they will be referred to as STD (for standard), OKA (for OKAPI) and SMO (for smoothing function). We have also used one – called SMOL – in which the natural logarithm of the smoothing function was used, as this gives a more even spread of probability scores [28].

We have used two of the *bel*(*q*) belief functions from InQuery, specifically the SUM and WSUM operators. If *p*_1_, *p*_2_,..., *p*_*n *_represent the beliefs at the parent nodes of *q *with corresponding weights *w*_1_, *w*_2_...., *w*_*n *_then the belief at *q *is given by

In the SUM model, the database structure nodes are denoted by *d*_1_, *d*_2_,..., *d*_*l *_where *l *is the number of molecules in the database. The second layer of nodes corresponds to the fragments that are set in the fingerprint for the reference structure, and which are hence expected to be present in active molecules (by the similar property principle). The fragments are denoted in the figure by *r*_1_, *r*_2_,..., *r*_*k*_, where *k *is the number of non-zero features set in the reference structure's fingerprint. In order to get a probability score for one molecule a SUM-operator is used, which combines the partial beliefs of the posterior probabilities into a single score for each database structure. The SUM operator takes account only of the presence or absence of each fragment in the reference structure's fingerprint; the WSUM operator additionally uses the number of occurrences of each such fragment as a weight in the calculation of the probability for each database structure.

### Testing

The searches of the MDDR and WOMBAT databases (see EXPERIMENTAL) are presented in tables in Additional files 1 and 2, and 3 and 4, respectively. In each table we list the recall in terms of the mean and standard deviation for the percentage of the actives retrieved (when averaged over 20 searches for each activity class). Recall figures are presented for each of the four different belief functions in the two different networks, and the tables also contain the corresponding figures for Tanimoto (TAN) searches (which have been included in each right-most column for purposes of comparison). The bottom row of each table contains the mean values averaged over the complete set of activity classes. The results presented here are for the recall of the actives in the top-1% of the ranking, with the best-performing search method (i.e., that with the highest mean recall) bold-faced and italicized. Comparable sets of experiments were carried out using the top-5% of the rankings to evaluate the various searches; the relative performance of the various methods was unchanged and we have hence included the results only for the top-1%. Similar comments apply to experiments in which we evaluated the various methods in terms of the recall of active Murcko scaffolds [29], rather than of active molecules.

We consider first the sets of BIN results in Additional files 1 and 2 to determine the relative performance of the eight methods. The significance, if any, of the differences in performance was tested with Kendall's *W *test of statistical significance, which is used to evaluate the consistency of *k *different sets of ranked judgments of the same set of *N *different objects [30]. Here, we have considered each of the eleven activity classes as a judge ranking the eight different combinations of network and belief function in order of decreasing effectiveness (as measured by the mean recall), i.e., *k *= 11 and *N *= 8. Converting the values in Tables S1 and S2 (Additional files 1 and 2) to ranks, the computed value for *W *is 0.520. The significance of this value can be tested using the χ^2 ^distribution since, for *N *> 7,

with *N*-1 degrees of freedom. This yields a value for χ^2 ^that is highly significant (p < 0.0001). Given that a significant level of agreement has been achieved, Siegel and Castellan suggest that the best overall ranking of the *N *objects can be obtained using their mean ranks averaged over the *k *judges [30]. This yields the following ranking for the MDDR database:

WSUM-OKA > SUM-OKA > WSUM-SMOL > SUM-SMOL > WSUM-STD > WSUM-SMO > SUM-SMO > SUM-STD

An entirely comparable analysis for the eight sets of BIN results in Tables S3 and S4 (Additional files 3 and 4) yields a value for *W *of 0.527; this is again highly significant and yields the following ranking for the WOMBAT database:

SUM-OKA > SUM-SMOL > WSUM-SMOL > WSUM-SMO > SUM-STD > SUM-SMO > WSUM-OKA > WSUM-STD

Both of these rankings are in broad accord with the mean recall values in the bottom rows of Tables S1-S4 (Additional files 1, 2, 3, 4).

SUM-OKA has performed well in both datasets, and we have hence used this function to determine whether there is any significant difference between the effectiveness of BIN-based and TAN-based searching. The difference has been assessed using the Sign test, a non-parametric test that is applicable to sets of paired observations such as these [30]. Specifically, assume that we have *N *pairs of observations – in this case the mean recall figures for each of the activity classes for SUM-OKA and for TAN – where there is a difference in the observed values; assume further that the first method performs better on *x *occasions (and hence that the second performs better on *N-x *occasions). The Sign test uses the binomial formula to check whether min{*x*, *N-x*} would have been expected to have occurred by chance if the two possible outcomes were equally likely; if this is not the case then we can assume that the two methods are significantly different (two-tailed test) or that one method is significantly better than the other (one-tailed test). For the MDDR data, SUM-OKA outperforms TAN 9 times out of 11, for which the one-tailed significance value is 0.033, i.e., a significant difference (*p *< 0.05); for the WOMBAT data, SUM-OKA outperforms TAN 13 times out of 14, for which the one-tailed significance value is 0.001, i.e., again a highly significant difference.

We can obtain further insights into the relative performance of the BIN and TAN searches if we consider the degree of structural diversity in the activity classes. The classes in Tables S1-S4 (Additional files 1, 2, 3, 4) have been listed in order of decreasing structural diversity (see EXPERIMENTAL), and it will be seen that TAN is the best method over all the nine methods for the two most heterogeneous classes in Tables S1 and S2 (Additional files 1 and 2) (the protein kinase C and cyclooxygenase inhibitors). TAN is also the best method overall for the most heterogeneous class in Tables S3 and S4 (Additional files 3 and 4) (again the cyclooxygenase inhibitors). This suggests that the relative performance of the two approaches – BIN as represented by SUM-OKA and TAN – depends on the nature of the active molecules that are being sought.

We have investigated this suggestion by carrying out screening experiments using the ten homogeneous classes in MDDR-HOM and the ten heterogeneous classes in MDDR-HET (see EXPERIMENTAL). The results of these searches are presented in the tables in Additional files 5 and 6, and 7 and 8, respectively. Inspection of these tables shows that SUM-OKA (and also SUM-SMOL) performs very well for the homogeneous activity classes and very poorly for the heterogeneous classes, and *vice versa *for TAN. The results in these tables hence provide strong evidence for the belief that Bayesian inference networks are noticeably less effective when there is a high level of structural diversity in the actives that are to be retrieved. Thus, if we consider MDDR-HOM, SUM-OKA outperforms TAN for nine of the ten activity classes, for which the one-tailed Sign test significance value is 0.011; whereas for MDDR-HET, the situation is completely reversed, with TAN outperforming SUM-OKA for nine of the ten activity classes.

## Discussion

We draw two principal conclusions from the experimental results presented in Tables S1-S8 (Additional files 1, 2, 3, 4, 5, 6, 7, 8). First, that BIN, specifically using the SUM-OKA belief function, is significantly superior to TAN when averaged over a range of different activity classes. Second, that TAN is significantly superior to BIN when attention is focused on structurally diverse activity classes.

It is not clear why there is such a marked difference in behaviour between BIN and TAN when different types of dataset are screened. In previous work, we have established the importance of molecular size in similarity searching using different types of similarity coefficient [31], and this may play a role here. Specifically, the mean molecular weights for the MDDR-HOM and MDDR-HET activity classes were 541.2 and 332.7, and this difference is reflected in the following related parameters: mean number of H-bond acceptors (8.9 and 4.0), mean number of H-bond donors (4.3 and 1.4), and mean number of bits set in the fingerprint (88.7 and 55.9). In this context, it is interesting to note that (when used for text retrieval purposes) the OKA belief function contains a length-normalisation term to minimize the bias of the STD belief function towards the retrieval of longer documents (i.e., larger molecules in the present context) [18]; it may be that an alternative normalization would be appropriate here. Whatever the reason, the relatively poor performance of SUM-OKA (and the other types of BIN search) on the diverse sets of actives is an undesirable characteristic of the BIN approach, since this would appear to lessen its attractiveness for scaffold hopping, one of the most important functions of an effective system for virtual screening.

To probe further the differences in the BIN and TAN searches, we compared the sets of actives retrieved in the two types of search. This comparison (again using SUM-OKA to represent BIN) is shown in Tables 1 and 2, which summarise the mean degree of overlap in the search outputs, when averaged over the twenty searches for each of the activity classes. The figures listed in Table 1 are the percentage (mean and standard deviation) of the active molecules retrieved by both SUM-OKA and TAN or by only one of these two search methods. It will be seen that SUM-OKA retrieves more unique actives than does TAN for the MDDR, MDDR-HOM and WOMBAT datasets, with the converse applying for the MDDR-HET dataset. To put these figures in context, Table 2 presents the same data in terms of the actual numbers of retrieved actives (rather than percentages). Thus, for a typical WOMBAT search, SUMO-OKA and TAN searches would result in 122.80 actives that were common to both top-1% lists, 56.37 actives that were unique to the SUM-OKA list and 26.84 actives that were unique to the TAN list. There did not appear to be any marked differences in the sizes of the active molecules retrieved by the two approaches.

**Table 1:** Overlap of actives (mean and standard deviation) in the top-1% search outputs from SUM-OKA and TAN searches: percentage of active molecules retrieved by both SUM-OKA and TAN ("Overlap") or only by one of these two search methods.

	Percentage of actives					
	
Dataset	Overlap		SUM-OKA		TAN	
MDDR	14.81	12.67	6.58	4.28	4.40	2.23
MDDR-HOM	69.21	18.48	13.39	8.84	3.58	3.75
MDDR-HET	7.05	6.36	2.38	1.86	4.71	3.75
WOMBAT	22.37	16.01	8.43	5.38	3.84	2.01

**Table 2:** Overlap of actives (mean and standard deviation) in the top-1% search outputs from SUM-OKA and TAN searches: number of active molecules retrieved by both SUM-OKA and TAN ("Overlap") or only by one of these two search methods.

	Number of actives					
	
Dataset	Overlap		SUM-OKA		TAN	
MDDR	131.58	154.10	56.41	47.71	35.89	27.40
MDDR-HOM	234.54	262.74	63.53	91.37	21.39	42.69
MDDR-HET	44.17	35.17	14.89	8.52	32.56	29.79
WOMBAT	122.80	71.39	56.37	44.62	26.84	21.05

A search of SciFinder Scholar in January 2009 revealed 107 references to Bayesian inference networks, mostly relating to gene expression and regulation and to analytical chemistry. and without any relating to applications in chemoinformatics. While this paper was being prepared for submission, we became aware of the work of Abdo and Salim [32], who have very recently described experiments with MDDR data that are similar to some of those reported here. Specifically, they carried out searches for a set of twelve activity classes, nine of which overlap with those in Tables S1 and S2 (Additional files 1 and 2), using a BIN that was based on the WSUM-OKA combination and EHFC_4, EEFC_4, ECFC_4, FHFC_4, FEFC_4 and FCFC_4 fingerprints. The principal difference between their experiments and ours is the composition of the database that was used for their experiments. We used the set of 102 K MDDR structures and eleven associated activity classes that have been used in several previous virtual screening studies (as well as the MDDR-HOM, MDDR-HET and WOMBAT datasets); however Abdo and Salim use a small subset of the MDDR database, containing just 40 K structures. They concluded that BIN out-performed TAN-based searching whilst noting, like us, that the BIN performance was affected by the diversity of the active molecules that were being sought. Their conclusion as to the overall superiority of BIN is based on their Table S5 (Additional file 5), which compares the recall for the best BIN searches (based on the EHFC_4 fingerprint) with the TAN recall; the former does better for eight of the twelve activity classes and latter does better for the other four; however, a one-tailed Sign test on this data shows that the differences in performance for these data are not statistically significant (*p *= 0.194). Our experiments, conversely, have demonstrated the significant superiority of the BIN approach when a range of types of activity class is studied.

## Conclusion

In this paper, we have evaluated the use of Bayesian inference networks for the implementation of similarity-based virtual screening. Our experiments with the MDDR and WOMBAT databases show that the networks provide an effective tool for ligand-based virtual screening. Specifically, our experiments have demonstrated the significant superiority of the best of the methods – referred to here as SUM-OKA -for screening a range of types of activity class when compared to a conventional screening system based on the Tanimoto coefficient. However, Tanimoto-based screening is significantly more effective if attention is focused on the more challenging task of identifying structurally diverse sets of active molecules; this might limit the effectiveness of the BIN approach for scaffold-hopping applications.

The search results presented here, in particular those in Tables 1 and 2, provide some evidence for the belief that it would be beneficial to combine the search outputs from BIN-based and TAN-based screening. Future work will hence consider the use of data fusion methods to combine these two approaches [33]. It is also our intention to use data fusion to combine the results of BIN searches using multiple reference structures since it is easy to extend a network to incorporate different sources of evidence (such as that from different reference structures). We also hope to study further the effect of structural diversity on the relative effectiveness of BIN and TAN searching.

### Experimental

Our experiments have used two of the most popular chemoinformatics databases: the MDL Drug Data Report database (MDDR, available from Symyx Technologies at http://www.symyx.com/products/databases/bioactivity/mddr/index.jsp) and the *World of Molecular Bioactivity *database (WOMBAT, available from Sunset Molecular Discovery LLC at http://www.sunsetmolecular.com/). The version of MDDR used here was that originally described by Hert *et al*. and used subsequently, by both us and others, for the validation of virtual screening methods [34-36]. It contains 102,516 molecules, with searches being carried out not only for the original eleven activity classes described by Hert et al. but also for two additional sets of activity classes: one chosen to be as structurally homogeneous as possible (MDDR-HOM) and one chosen to be as structurally heterogeneous as possible (MDDR-HET) [37]. The three sets of MDDR classes are listed in Tables 3, 4 and 5. Each row of the table contains an activity class, the number of molecules belonging to the class, and the class's diversity, this being based on the pair-wise Tanimoto similarities calculated using the standard Unity 2D fingerprint (available from Tripos Inc. at http://www.tripos.com). The version of WOMBAT used here contained 138,127 molecules with searches being carried out for the 14 activity classes listed in Table 6. The identification of these classes is described in detail by Gardiner *et al*. [38].

**Table 3:** MDDR activity classes used in the virtual screening experiments.

Activity class	Active molecules	Pairwise similarity	
Renin inhibitors	1130	0.573	0.11
HIV protease inhibitors	750	0.446	0.12
Thrombin inhibitors	803	0.419	0.13
Angiotensin II AT1 antagonists	943	0.403	0.10
Substance P antagonists	1246	0.399	0.11
5HT3 antagonists	752	0.351	0.12
5HT reuptake inhibitors	359	0.345	0.12
D2 antagonists	395	0.345	0.10
5HT1A agonists	827	0.343	0.10
Protein kinase C inhibitors	453	0.323	0.14
Cyclooxygenase inhibitors	636	0.268	0.09

The three columns in each table give the number of actives in the class and the mean and the standard deviation of the inter-molecular similarities for all the molecules in the chosen class.

**Table 4:** MDDR-HOM activity classes used in the virtual screening experiments.

Activity class	Active molecules	Pairwise similarity	
Muscarinic (M1) agonists	848	0.206	0.098
NMDA receptor antagonists	1311	0.199	0.090
Nitric oxide synthase inhibitors	377	0.189	0.086
Dopamine beta-hydroxylase inhibitors	95	0.229	0.076
Aldose reductase inhibitors	882	0.232	0.096
Reverse transcriptase inhibitors	519	0.218	0.095
Aromatase inhibitors	513	0.229	0.117
Cyclooxygenase inhibitors	636	0.220	0.107
Phospholipase A2 inhibitors	704	0.224	0.111
Lipoxygenase inhibitors	2555	0.224	0.110

Details as for Table 3.

**Table 5:** MDDR-HET activity classes used in the virtual screening experiments.

Activity class	Active molecules	Pairwise similarity	
Adenosine (A1) agonists	88	0.524	0.124
Adenosine (A2) agonists	71	0.536	0.137
Renin inhibitors	1130	0.459	0.119
CCK agonists	79	0.452	0.099
Monocyclic beta-lactams	76	0.549	0.084
Cephalosporins	1312	0.501	0.098
Carbacephems	73	0.487	0.099
Carbapenems	896	0.457	0.124
Tribactams	74	0.548	0.150
Vitamin D analogues	279	0.574	0.105

Details as for Table 3.

**Table 6:** WOMBAT activity classes used in the virtual screening experiments.

Activity class	Active molecules	Pairwise similarity	
Renin inhibitors	474	0.592	0.109
Protein kinase C inhibitors	142	0.565	0.277
Matrix metalloprotease inhibitors	694	0.444	0.148
Angiotensin II AT1 antagonists	724	0.443	0.131
HIV protease inhibitors	1128	0.442	0.146
Substance P antagonists	558	0.427	0.127
Thrombin inhibitors	421	0.418	0.144
5HT1A antagonists	592	0.399	0.134
Factor Xa inhibitors	842	0.394	0.124
5HT3 antagonists	220	0.377	0.175
Acetylcholine esterase inhibitors	503	0.373	0.155
D2 antagonists	910	0.367	0.116
Phosphodiesterase inhibitors	596	0.359	0.136
Cyclooxygenase inhibitors	965	0.324	0.139

Details as for Table 3.

The molecules in the two databases were characterised by ECFC_6 fingerprints. These encode circular substructures of radius three bonds centred on each of the non-hydrogen atoms in a molecule, and with each element in the fingerprint containing the number of times that a particular substructure occurred in a molecule. The 1024-element fingerprints were generated using the Pipeline Pilot software. Experiments were also carried out on the MDDR dataset using the ECFC_4, SCFC_4 and EHFC_4 fingerprints: performance was analogous to that obtained for ECFC_6, and the results have hence not been included here.

The belief functions that lie at the heart of a BIN all have parameters that have to be set. For the belief functions used here, these are *db *for the STD and OKA functions and λ for the SMO and SMOL functions (see above). The values used for the results reported in Tables S1-S8 (Additional files 1, 2, 3, 4, 5, 6, 7, 8) were *db *= 0.5 for STD, *db *= 0.2 for OKA, and λ = 0.6 for both SMO and SMOL. These values were chosen after initial, parameterisation runs, which showed that BIN performance was slightly affected by the precise choice of parameter value (runs with both parameters in the range 0.1–1.0 in steps of 0.1).

Many different criteria have been suggested for the evaluation of virtual-screening experiments [39-41]. The experiments reported here used the simplest such criterion: the recall, i.e., the percentage of the active molecules retrieved at some cut-off point in the ranking, for which we have used both the top-1% and the top-5% of the rankings. The top-5% results have not been included in the paper since they were analogous, in terms of the relative performance of the various methods, to those reported for the top-1%. Twenty randomly-selected molecules from each activity class were used in turn as the reference structure, and the search performance averaged over all of the reference structures for the class to obtain the mean and standard deviation; the final measure of search effectiveness was then obtained by averaging over the activity classes, so that each class contributed equally to the overall performance.

To provide a basis of comparison for the BIN searches, analogous experiments were carried out using a conventional similarity searching system (TAN) based on the full version of the Tanimoto coefficient [8]. For two molecular fingerprints *X *and *Y*, the similarity between the corresponding molecules is

where the summations are over all of the elements in each fingerprint, and where each element contains the frequency of occurrence of a substructural fragment. The use of frequencies of occurrence has been shown previously to enhance search effectiveness when compared to conventional similarity measures based on binary fingerprints [42-44].

## Competing interests

The authors declare that they have no competing interests.

## Authors' contributions

BC jointly conceived the study, and participated in its design and coordination; CM carried out all the experimental work; PW jointly conceived the study, participated in its design and coordination, and drafted the manuscript. All authors read and approved the final manuscript.

## Supplementary Material

Additional file 1 **Table S1. **Recall of actives in the top-1% of the ranked MDDR database using the Bayesian SUM inference network and Tanimoto searches. The belief functions used are STD (for the standard function used in the InQuery project), OKA (for that used in the OKAPI project), SMO (for the language-modeling smoothing function) and SMOL (for the natural logarithm of the smoothing function). Each pair of columns lists the mean and the standard deviation for the percentage recall. Click here for file

Additional file 2 **Table S2. **Recall of actives in the top-1% of the ranked MDDR database using the Bayesian WSUM inference network and Tanimoto searches. Details as for Additional file 1. Click here for file

Additional file 3 **Table S3. **Recall of actives in the top-1% of the ranked WOMBAT database using the Bayesian SUM inference network and Tanimoto searches. Details as for Additional file 1. Click here for file

Additional file 4 **Table S4. **Recall of actives in the top-1% of the ranked WOMBAT database using the Bayesian WSUM inference network and Tanimoto searches. Details as for Additional file 1. Click here for file

Additional file 5 **Table S5. **Recall of actives in the top-1% of the ranked MDDR-HOM database using the Bayesian SUM inference network and Tanimoto searches. Details as for Additional file 1. Click here for file

Additional file 6 **Table S6. **Recall of actives in the top-1% of the ranked MDDR-HOM database using the Bayesian WSUM inference network and Tanimoto searches. Details as for Additional file 1. Click here for file

Additional file 7 **Table S7. **Recall of actives in the top-1% of the ranked MDDR-HET database using the Bayesian SUM inference network and Tanimoto searches. Details as for Additional file 1. Click here for file

Additional file 8 **Table S8. **Recall of actives in the top-1% of the ranked MDDR-HET database using the Bayesian WSUM inference network and Tanimoto searches. Details as for Additional file 1. Click here for file
