# Supplementary material for: Evaluation of a Bayesian inference network for ligand-based virtual screening
Source: J Cheminform. 2009 Apr 29;1:5. doi: 10.1186/1758-2946-1-5 (PMC3225873; doi:10.1186/1758-2946-1-5)
Supplement: Additional file 1 — Table S1. Recall of actives in the top-1% of the ranked MDDR database using the Bayesian SUM inference network and Tanimoto searches. The belief functions used are STD (for the standard function used in the InQuery project), OKA (for that used in the OKAPI project), SMO (for the language-modeling smoothing function) and SMOL (for the natural logarithm of the smoothing function). Each pair of columns lists the mean and the standard deviation for the percentage recall. [file 1758-2946-1-5-S1.doc]

| Activity class | SUM | | | | | | | | TAN | |
| --- | --- | --- | --- | --- | --- | --- | --- | --- | --- | --- |
|  | STD | | OKA | | SMO | | SMOL | |  |  |
| Renin inhibitors | 56.30 | 9.86 | 61.09 | 11.49 | 34.54 | 11.79 | 61.27 | 11.22 | 56.69 | 19.38 |
| HIV protease inhibitors | 21.59 | 13.22 | 24.83 | 15.72 | 20.49 | 12.15 | ***25.47*** | 15.25 | 22.66 | 15.97 |
| Thrombin inhibitors | 15.27 | 7.26 | 19.35 | 10.08 | 12.20 | 7.26 | 18.28 | 8.81 | 12.54 | 8.63 |
| Angiotensin II AT1 antagonists | 40.23 | 8.64 | 41.31 | 7.00 | 32.40 | 10.49 | ***41.55*** | 7.07 | 35.22 | 9.54 |
| Substance P antagonists | 13.33 | 6.64 | ***18.49*** | 10.87 | 11.54 | 6.53 | 16.65 | 9.10 | 16.38 | 10.75 |
| 5HT3 antagonists | 10.39 | 6.64 | 15.74 | 9.33 | 10.85 | 6.90 | 14.12 | 8.41 | 13.13 | 8.59 |
| 5HT reuptake inhibitors | 9.62 | 8.82 | 10.74 | 8.84 | 7.91 | 6.48 | 10.93 | 9.07 | 10.04 | 7.54 |
| D2 antagonists | 7.61 | 4.85 | 9.30 | 5.74 | 7.15 | 4.79 | 8.84 | 5.63 | 8.75 | 4.95 |
| 5HT1A agonists | 10.73 | 7.30 | ***13.33*** | 8.19 | 10.63 | 6.07 | 12.56 | 7.87 | 12.10 | 5.91 |
| Protein kinase C inhibitors | 10.22 | 9.52 | 14.65 | 12.06 | 14.08 | 9.51 | 13.11 | 11.37 | ***16.47*** | 13.02 |
| Cyclooxygenase inhibitors | 4.72 | 2.89 | 7.20 | 4.45 | 8.13 | 5.08 | 6.39 | 3.85 | ***7.94*** | 4.58 |
| Mean | 18.18 | 15.90 | 21.46 | 16.13 | 15.45 | 9.64 | 20.83 | 16.53 | 19.27 | 14.63 |
