# Supplementary material for: Evaluation of a Bayesian inference network for ligand-based virtual screening
Source: J Cheminform. 2009 Apr 29;1:5. doi: 10.1186/1758-2946-1-5 (PMC3225873; doi:10.1186/1758-2946-1-5)
Supplement: Additional file 2 — Table S2. Recall of actives in the top-1% of the ranked MDDR database using the Bayesian WSUM inference network and Tanimoto searches. Details as for Additional file 1. [file 1758-2946-1-5-S2.doc]

| Activity class | WSUM | | | | | | | | TAN | |
| --- | --- | --- | --- | --- | --- | --- | --- | --- | --- | --- |
|  | STD | | OKA | | SMO | | SMOL | |  |  |
| Renin inhibitors | 60.41 | 9.76 | ***66.83*** | 11.83 | 58.06 | 14.38 | 64.64 | 12.93 | 56.69 | 19.38 |
| HIV protease inhibitors | 22.56 | 12.81 | 24.11 | 13.95 | 18.88 | 14.43 | 23.45 | 14.94 | 22.66 | 15.97 |
| Thrombin inhibitors | 18.98 | 11.77 | ***22.93*** | 13.47 | 11.31 | 8.29 | 20.73 | 12.77 | 12.54 | 8.63 |
| Angiotensin II AT1 antagonists | 38.81 | 8.23 | 41.38 | 8.00 | 31.33 | 10.87 | 39.84 | 8.62 | 35.22 | 9.54 |
| Substance P antagonists | 14.20 | 5.27 | 16.74 | 8.10 | 15.90 | 9.75 | 15.64 | 7.51 | 16.38 | 10.75 |
| 5HT3 antagonists | 13.50 | 9.38 | 17.44 | 9.00 | 12.87 | 7.48 | ***19.18*** | 9.88 | 13.13 | 8.59 |
| 5HT reuptake inhibitors | 10.81 | 8.63 | 11.20 | 9.67 | 9.54 | 7.42 | ***11.38*** | 9.71 | 10.04 | 7.54 |
| D2 antagonists | 9.78 | 6.23 | 10.33 | 6.52 | 7.72 | 4.57 | ***10.54*** | 6.22 | 8.75 | 4.95 |
| 5HT1A agonists | 9.98 | 5.89 | 12.88 | 7.78 | 11.09 | 5.32 | 12.91 | 7.91 | 12.10 | 5.91 |
| Protein kinase C inhibitors | 10.96 | 9.84 | 12.16 | 10.43 | 13.11 | 11.51 | 12.42 | 10.47 | ***16.47*** | 13.02 |
| Cyclooxygenase inhibitors | 5.24 | 3.66 | 6.59 | 4.60 | 6.73 | 4.75 | 7.29 | 4.85 | ***7.94*** | 4.58 |
| Mean | 19.57 | 16.30 | ***22.05*** | 17.63 | 17.87 | 14.95 | 21.64 | 16.80 | 19.27 | 14.63 |
