# Supplementary material for: Evaluation of a Bayesian inference network for ligand-based virtual screening
Source: J Cheminform. 2009 Apr 29;1:5. doi: 10.1186/1758-2946-1-5 (PMC3225873; doi:10.1186/1758-2946-1-5)
Supplement: Additional file 3 — Table S3. Recall of actives in the top-1% of the ranked WOMBAT database using the Bayesian SUM inference network and Tanimoto searches. Details as for Additional file 1. [file 1758-2946-1-5-S3.doc]

| Activity class | SUM | | | | | | | | TAN | |
| --- | --- | --- | --- | --- | --- | --- | --- | --- | --- | --- |
|  | STD | | OKA | | SMO | | SMOL | |  |  |
| Renin inhibitors | 51.05 | 23.79 | ***72.27*** | 23.61 | 29.3 | 13.37 | 66.65 | 24.24 | 61.1 | 29.26 |
| Protein kinase C inhibitors | 57.29 | 31.15 | ***59.86*** | 33.21 | 58.03 | 32.01 | 58.63 | 32.46 | 59.26 | 32.87 |
| Matrix metalloprotease inhibitors | 20.96 | 15.11 | ***26.04*** | 16.56 | 22.12 | 16.27 | 25.21 | 16.30 | 21.31 | 14.79 |
| Angiotensin II AT1 antagonists | 39.27 | 16.49 | 52.15 | 18.29 | 24.78 | 12.05 | ***53.36*** | 18.71 | 32.8 | 14.34 |
| HIV protease inhibitors | 18.71 | 10.15 | 28.10 | 13.40 | 19.05 | 7.46 | ***28.75*** | 13.60 | 21.25 | 12.34 |
| Substance P antagonists | 19.00 | 10.88 | ***30.04*** | 14.97 | 22.42 | 12.80 | 28.66 | 14.18 | 25.37 | 16.47 |
| Thrombin inhibitors | 10.39 | 6.62 | 20.63 | 10.98 | 13.49 | 8.92 | 17.07 | 9.41 | 19.31 | 10.93 |
| 5HT1A antagonists | 15.46 | 8.96 | ***22.42*** | 13.77 | 15.26 | 8.94 | 20.95 | 12.32 | 21.17 | 12.66 |
| Factor Xa inhibitors | 18.08 | 11.00 | ***21.03*** | 10.97 | 15.48 | 8.98 | 20.93 | 11.25 | 19.14 | 9.02 |
| 5HT3 antagonists | 19.25 | 11.47 | ***22.39*** | 11.53 | 19.14 | 11.38 | 21.55 | 11.59 | 20.86 | 10.86 |
| Acetylcholine esterase inhibitors | 12.14 | 6.02 | ***16.10*** | 8.37 | 8.99 | 3.36 | 15.21 | 8.00 | 10.29 | 3.36 |
| D2 antagonists | 9.70 | 5.69 | ***14.21*** | 8.34 | 10.9 | 5.86 | 12.94 | 7.79 | 13.43 | 8.10 |
| Phosphodiesterase inhibitors | 15.61 | 7.89 | ***19.77*** | 8.95 | 13.98 | 6.76 | 18.45 | 8.93 | 13.28 | 7.50 |
| Cyclooxygenase inhibitors | 17.96 | 13.74 | 27.37 | 18.48 | 28.49 | 18.85 | 25.77 | 17.59 | ***29.85*** | 21.66 |
| Mean | 23.21 | 14.92 | ***30.88*** | 17.57 | 21.53 | 12.19 | 29.58 | 17.09 | 26.31 | 15.57 |
