# Supplementary material for: Evaluation of a Bayesian inference network for ligand-based virtual screening
Source: J Cheminform. 2009 Apr 29;1:5. doi: 10.1186/1758-2946-1-5 (PMC3225873; doi:10.1186/1758-2946-1-5)
Supplement: Additional file 4 — Table S4. Recall of actives in the top-1% of the ranked WOMBAT database using the Bayesian WSUM inference network and Tanimoto searches. Details as for Additional file 1. [file 1758-2946-1-5-S4.doc]

| Activity class | WSUM | | | | | | | | TAN | |
| --- | --- | --- | --- | --- | --- | --- | --- | --- | --- | --- |
|  | STD | | OKA | | SMO | | SMOL | |  |  |
| Renin inhibitors | 40.66 | 19.41 | 49.77 | 15.89 | 59.16 | 27.28 | 55.37 | 16.59 | 61.1 | 29.26 |
| Protein kinase C inhibitors | 54.51 | 31.65 | 36.48 | 23.75 | 59.12 | 32.71 | 43.59 | 27.05 | 59.26 | 32.87 |
| Matrix metalloprotease inhibitors | 13.29 | 6.50 | 16.12 | 8.93 | 17.32 | 11.03 | 17.19 | 9.96 | 21.31 | 14.79 |
| Angiotensin II AT1 antagonists | 29.09 | 13.69 | 23.67 | 10.51 | 28.65 | 14.52 | 26.57 | 11.41 | 32.8 | 14.34 |
| HIV protease inhibitors | 18.43 | 8.18 | 18.35 | 7.21 | 17.32 | 10.70 | 19.02 | 8.21 | 21.25 | 12.34 |
| Substance P antagonists | 15.01 | 4.80 | 17.31 | 7.07 | 24.44 | 14.71 | 19.83 | 8.93 | 25.37 | 16.47 |
| Thrombin inhibitors | 17.57 | 9.82 | 21.79 | 11.05 | 15.63 | 7.47 | ***22.51*** | 11.35 | 19.31 | 10.93 |
| 5HT1A antagonists | 17.73 | 14.04 | 20.14 | 16.11 | 22.08 | 12.17 | 21.85 | 16.02 | 21.17 | 12.66 |
| Factor Xa inhibitors | 14.63 | 8.83 | 14.17 | 8.99 | 14.38 | 8.57 | 16.16 | 10.39 | 19.14 | 9.02 |
| 5HT3 antagonists | 19.00 | 10.76 | 18.48 | 11.04 | 18.73 | 9.26 | 19.98 | 11.04 | 20.86 | 10.86 |
| Acetylcholine esterase inhibitors | 8.55 | 4.55 | 9.28 | 4.04 | 11.09 | 3.99 | 9.68 | 3.86 | 10.29 | 3.36 |
| D2 antagonists | 11.72 | 7.75 | 12.25 | 6.52 | 12.19 | 7.26 | 13.23 | 7.09 | 13.43 | 8.10 |
| Phosphodiesterase inhibitors | 9.96 | 3.71 | 11.71 | 5.38 | 12.47 | 6.57 | 12.38 | 5.56 | 13.28 | 7.50 |
| Cyclooxygenase inhibitors | 18.1 | 15.84 | 22.89 | 17.41 | 23.27 | 17.71 | 26.76 | 20.12 | ***29.85*** | 21.66 |
| Mean | 20.59 | 12.75 | 20.89 | 10.66 | 23.99 | 15.72 | 23.15 | 12.41 | 26.31 | 15.57 |
