# Supplementary material for: Evaluation of a Bayesian inference network for ligand-based virtual screening
Source: J Cheminform. 2009 Apr 29;1:5. doi: 10.1186/1758-2946-1-5 (PMC3225873; doi:10.1186/1758-2946-1-5)
Supplement: Additional file 5 — Table S5. Recall of actives in the top-1% of the ranked MDDR-HOM database using the Bayesian SUM inference network and Tanimoto searches. Details as for Additional file 1. [file 1758-2946-1-5-S5.doc]

| Activity class | SUM | | | | | | | | TAN | |
| --- | --- | --- | --- | --- | --- | --- | --- | --- | --- | --- |
|  | STD | | OKA | | SMO | | SMOL | |  |  |
| Adenosine (A1) agonists | 96.93 | 0.65 | ***98.07*** | 1.28 | 84.89 | 14.47 | 97.61 | 0.97 | 80.85 | 26.15 |
| Adenosine (A2) agonists | 95.35 | 2.74 | ***96.97*** | 2.63 | 81.06 | 9.80 | 96.97 | 2.55 | 76.13 | 10.78 |
| Renin inhibitors | 56.3 | 9.86 | 61.09 | 11.49 | 34.54 | 11.79 | 61.27 | 11.22 | 56.69 | 19.38 |
| CCK agonists | ***58.42*** | 6.01 | 54.37 | 8.37 | 44.87 | 9.55 | 58.35 | 6.58 | 57.85 | 5.53 |
| Monocyclic beta-lactams | 93.55 | 2.73 | 96.71 | 2.68 | ***96.84*** | 3.18 | 95.79 | 2.47 | 93.42 | 1.35 |
| Cephalosporins | 75.20 | 1.70 | 75.39 | 1.40 | 60.48 | 18.60 | ***75.47*** | 1.34 | 64.86 | 13.39 |
| Carbacephems | 69.04 | 16.33 | ***74.04*** | 17.08 | 67.33 | 13.03 | 71.85 | 18.45 | 73.90 | 15.51 |
| Carbapenems | 79.60 | 8.78 | ***87.41*** | 4.49 | 60.67 | 10.58 | 87.18 | 5.16 | 61.17 | 7.88 |
| Tribactams | 99.66 | 1.23 | ***100.00*** | 0.00 | 94.86 | 4.99 | ***100.00*** | 0.00 | 79.05 | 14.60 |
| Vitamin D analogous | 99.09 | 0.96 | ***99.64*** | 0.00 | 98.78 | 1.08 | ***99.64*** | 0.00 | 96.33 | 0.83 |
| Mean | 82.31 | 16.91 | 84.37 | 17.04 | 72.43 | 22.42 | ***84.41*** | 16.32 | 74.03 | 13.99 |
