# Supplementary material for: Evaluation of a Bayesian inference network for ligand-based virtual screening
Source: J Cheminform. 2009 Apr 29;1:5. doi: 10.1186/1758-2946-1-5 (PMC3225873; doi:10.1186/1758-2946-1-5)
Supplement: Additional file 6 — Table S6. Recall of actives in the top-1% of the ranked MDDR-HOM database using the Bayesian WSUM inference network and Tanimoto searches. Details as for Additional file 1. [file 1758-2946-1-5-S6.doc]

| Activity class | WSUM | | | | | | | | TAN | |
| --- | --- | --- | --- | --- | --- | --- | --- | --- | --- | --- |
|  | STD | | OKA | | SMO | | SMOL | |  |  |
| Adenosine (A1) agonists | 56.76 | 17.44 | 62.95 | 13.02 | 75.97 | 26.61 | 74.09 | 13.58 | 80.85 | 26.15 |
| Adenosine (A2) agonists | 61.90 | 12.18 | 63.45 | 16.64 | 76.06 | 19.44 | 70.21 | 15.80 | 76.13 | 10.78 |
| Renin inhibitors | 60.41 | 9.76 | ***66.83*** | 11.83 | 58.06 | 14.38 | 64.64 | 12.93 | 56.69 | 19.38 |
| CCK agonists | 48.16 | 14.96 | 41.96 | 15.90 | 58.04 | 7.89 | 44.75 | 16.45 | 57.85 | 5.53 |
| Monocyclic beta-lactams | 60.92 | 18.47 | 59.01 | 10.22 | 87.57 | 3.83 | 71.05 | 9.57 | 93.42 | 1.35 |
| Cephalosporins | 41.08 | 15.14 | 38.87 | 16.93 | 67.28 | 11.11 | 47.31 | 19.05 | 64.86 | 13.39 |
| Carbacephems | 56.51 | 12.47 | 58.29 | 14.85 | 61.99 | 15.95 | 62.53 | 14.52 | 73.90 | 15.51 |
| Carbapenems | 34.30 | 11.21 | 37.79 | 6.99 | 61.19 | 12.99 | 41.98 | 9.74 | 61.17 | 7.88 |
| Tribactams | 40.34 | 7.06 | 53.72 | 12.83 | 69.80 | 25.17 | 60.95 | 13.29 | 79.05 | 14.60 |
| Vitamin D analogous | 91.47 | 1.68 | 90.50 | 2.65 | 87.76 | 10.42 | 94.96 | 1.11 | 96.33 | 0.83 |
| Mean | 55.19 | 16.09 | 57.34 | 15.76 | 70.37 | 11.20 | 63.25 | 15.95 | 74.03 | 13.99 |
