# Supplementary material for: Evaluation of a Bayesian inference network for ligand-based virtual screening
Source: J Cheminform. 2009 Apr 29;1:5. doi: 10.1186/1758-2946-1-5 (PMC3225873; doi:10.1186/1758-2946-1-5)
Supplement: Additional file 7 — Table S7. Recall of actives in the top-1% of the ranked MDDR-HET database using the Bayesian SUM inference network and Tanimoto searches. Details as for Additional file 1. [file 1758-2946-1-5-S7.doc]

| Activity class | SUM | | | | | | | | TAN | |
| --- | --- | --- | --- | --- | --- | --- | --- | --- | --- | --- |
|  | STD | | OKA | | SMO | | SMOL | |  |  |
| Muscarinic (M1) agonists | 7.08 | 6.11 | 14.25 | 8.37 | 19.22 | 11.47 | 10.92 | 7.83 | ***24.16*** | 12.84 |
| NMDA receptor antagonists | 2.46 | 2.07 | 5.09 | 4.70 | 6.18 | 5.25 | 4.28 | 4.10 | ***7.40*** | 5.52 |
| Nitric oxide synthase inhibitors | 3.89 | 2.54 | 7.94 | 3.17 | 8.57 | 2.95 | 6.62 | 3.07 | ***8.78*** | 4.55 |
| Dopamine beta-hydroxylase inhibitors | 14.95 | 8.50 | 23.37 | 11.37 | 26.53 | 12.36 | 20.95 | 10.78 | ***26.58*** | 10.57 |
| Aldose reductase inhibitors | 6.77 | 4.41 | 9.16 | 4.38 | 9.29 | 4.58 | 8.92 | 4.34 | ***10.55*** | 4.77 |
| Reverse transcriptase inhibitors | 2.59 | 1.05 | ***3.03*** | 1.30 | 2.38 | 0.77 | 2.92 | 1.26 | 2.21 | 1.23 |
| Aromatase inhibitors | 16.22 | 12.51 | 20.59 | 14.11 | 20.9 | 13.63 | 19.26 | 13.51 | ***23.75*** | 15.25 |
| Cyclooxygenase inhibitors | 4.72 | 2.89 | 7.2 | 4.45 | ***8.13*** | 5.08 | 6.39 | 3.85 | 7.94 | 4.58 |
| Phospholipase A2 inhibitors | 2.39 | 1.96 | 3.49 | 3.58 | ***5.78*** | 5.98 | 3.12 | 2.90 | 4.75 | 5.09 |
| Lipoxygenase inhibitors | 2.37 | 2.49 | 3.66 | 2.83 | 3.94 | 2.66 | 3.29 | 2.72 | ***4.29*** | 2.56 |
| Mean | 6.34 | 5.18 | 9.78 | 7.28 | 11.09 | 8.15 | 8.67 | 6.58 | ***12.04*** | 9.17 |
