# Supplementary material for: Evaluation of a Bayesian inference network for ligand-based virtual screening
Source: J Cheminform. 2009 Apr 29;1:5. doi: 10.1186/1758-2946-1-5 (PMC3225873; doi:10.1186/1758-2946-1-5)
Supplement: Additional file 8 — Table S8. Recall of actives in the top-1% of the ranked MDDR-HET database using the Bayesian WSUM inference network and Tanimoto searches. Details as for Additional file 1. [file 1758-2946-1-5-S8.doc]

| Activity class | WSUM | | | | | | | | TAN | |
| --- | --- | --- | --- | --- | --- | --- | --- | --- | --- | --- |
|  | STD | | OKA | | SMO | | SMOL | |  |  |
| Muscarinic (M1) agonists | 11.00 | 6.54 | 18.87 | 12.00 | 19.2 | 10.24 | 18.84 | 10.73 | ***24.16*** | 12.84 |
| NMDA receptor antagonists | 3.05 | 2.33 | 4.79 | 2.64 | 5.34 | 4.40 | 5.48 | 3.29 | ***7.40*** | 5.52 |
| Nitric oxide synthase inhibitors | 6.31 | 3.39 | 8.04 | 3.49 | 6.87 | 3.70 | 8.29 | 3.60 | ***8.78*** | 4.55 |
| Dopamine beta-hydroxylase inhibitors | 20.84 | 10.86 | 20.53 | 12.31 | 15.53 | 8.26 | 20.16 | 10.58 | ***26.58*** | 10.57 |
| Aldose reductase inhibitors | 7.01 | 3.65 | 8.49 | 2.94 | 9.19 | 5.12 | 8.69 | 3.21 | ***10.55*** | 4.77 |
| Reverse transcriptase inhibitors | 2.01 | 1.17 | 2.06 | 1.10 | 2.43 | 1.35 | 2.05 | 1.27 | 2.21 | 1.23 |
| Aromatase inhibitors | 13.11 | 8.08 | 21.35 | 13.68 | 21.98 | 14.63 | 21.77 | 14.37 | ***23.75*** | 15.25 |
| Cyclooxygenase inhibitors | 5.24 | 3.66 | 6.59 | 4.60 | 6.73 | 4.75 | 7.29 | 4.85 | 7.94 | 4.58 |
| Phospholipase A2 inhibitors | 2.00 | 2.25 | 2.61 | 2.02 | 3.46 | 4.29 | 2.70 | 2.04 | 4.75 | 5.09 |
| Lipoxygenase inhibitors | 3.35 | 2.08 | 3.23 | 1.72 | 3.65 | 2.55 | 3.24 | 1.57 | ***4.29*** | 2.56 |
| Mean | 7.39 | 6.02 | 9.66 | 7.64 | 9.43 | 6.98 | 9.85 | 7.56 | ***12.04*** | 9.17 |
